# Complete Recovery From Acute Peroneal Nerve Palsy With Neurapraxia After Prolonged Cross-Legged Sitting: Successful Conservative Management of a Foot Drop and a Brief Review of the Literature

**DOI:** 10.7759/cureus.78465

**Published:** 2025-02-03

**Authors:** Muhammed Yusuf Afacan, Burak Ozturk, Derya Akbaba, Ahmed Necati Sahin, Mahmut Kürsat Ozsahin

**Affiliations:** 1 Department of Orthopedics and Traumatology, Cerrahpasa Faculty of Medicine, Istanbul University-Cerrahpasa, Istanbul, TUR; 2 Department of Anatomy, Institute of Graduate Studies, Istanbul University-Cerrahpasa, Istanbul, TUR; 3 Cerrahpasa Faculty of Medicine, Istanbul University-Cerrahpasa, Istanbul, TUR

**Keywords:** common peroneal neuropathy, conservative treatment, cross-leg sitting, drop foot, neurapraxia, peripheral entrapment neuropathy, peroneal nerve palsy

## Abstract

Peroneal nerve palsy is the most common entrapment neuropathy of the lower extremity, often presenting with foot drop and sensory deficits. While trauma and space-occupying lesions are well-documented causes, prolonged static postures, such as cross-legged sitting, can lead to neurapraxia, a mere myelin injury, and a reversible conduction block caused by nerve compression. This case report aims to present the clinical course and successful conservative management of peroneal nerve palsy with foot drop in a 26-year-old male following prolonged cross-legged sitting, highlighting the unusual symptom presentation where typical nerve compression signs such as tingling, neuropathic pain, heaviness, or numbness were absent until the patient stood up. It also emphasizes the rare posture-related etiology, complete recovery without surgical intervention, and reviews similar rare cases to enhance clinical recognition of positional nerve compression syndromes.

A 26-year-old male developed acute foot drop and numbness in the right foot after sitting cross-legged on a hard surface for 2-3 hours without changing position. Physical examination revealed 0/5 strength in ankle dorsiflexion, hypoesthesia in the first web space, and steppage gait, with no history of trauma or prior symptoms. Radiographs excluded structural abnormalities. Conservative management, including an ankle-foot orthosis (AFO) and daily supplementation with neurotrophic agents (B vitamins, vitamin C, vitamin D3, zinc, and magnesium), was initiated. At the two-week follow-up, dorsiflexion strength improved to 3/5. By the one-month follow-up, the patient achieved complete recovery, with full restoration of muscle strength as 5/5, sensory function, and resolution of neuropathy. Acute peroneal nerve palsy with neurapraxia can result from prolonged cross-legged sitting due to compression at the fibular head. Conservative management, including neurotrophic supplementation and the use of an AFO, can achieve complete recovery without the need for surgical intervention. Early recognition, detailed patient history, and individualized treatment plans are essential for optimal outcomes. Surgical decompression should be considered judiciously in resistant cases based on the severity and progression of symptoms.

## Introduction

Peroneal nerve palsy is the most common entrapment neuropathy of the lower extremity, ranking third in frequency after ulnar and median neuropathies [1]. It most commonly occurs due to local compression at the level of the fibular head [2]. Other common compression sites of the peroneal nerve after branching include the anterior tarsal tunnel for the deep peroneal nerve and the deep crural fascia between the peroneal muscles for the superficial peroneal nerve [3]. Cases of foot drop developing after sitting in a cross-legged position have been reported in the literature [4]. In cases of atraumatic sudden onset foot drop, space-occupying lesions surrounding the peroneal nerve should be considered [5]. On physical examination, individuals with foot drops exhibit an abnormal gait pattern known as steppage gait, characterized by the absence of ankle dorsiflexion, which affects both the stance and swing phases of walking [6]. Foot drop is the most common symptom following peroneal nerve palsy [7]. Although some studies advocate for early surgical intervention regardless of the etiology, the majority of the literature cases recommended an initial conservative approach to treatment [8]. Management should involve identifying the underlying etiology and planning an appropriate rehabilitation process. This case report aims to present the clinical course and management of a 26-year-old male patient who developed peroneal nerve palsy with foot drop following prolonged cross-legged sitting. The case highlights an unusual presentation of symptoms, where the patient did not initially report typical nerve compression signs such as tingling, neuropathic pain, heaviness, or numbness until standing up. This report emphasizes the rare etiology related to the specific posture, underlining the clinical relevance of how prolonged cross-legged sitting can lead to nerve compression. The case also demonstrates the successful conservative management of neurapraxia (involving merely myelin injury) without the need for surgical intervention, resulting in complete recovery. Additionally, this report aims to review the literature on similar rare cases, contributing to a better understanding of positional nerve compression syndromes and helping practitioners recognize and manage such presentations effectively in clinical practice.

## Case presentation

**Figure 1:**
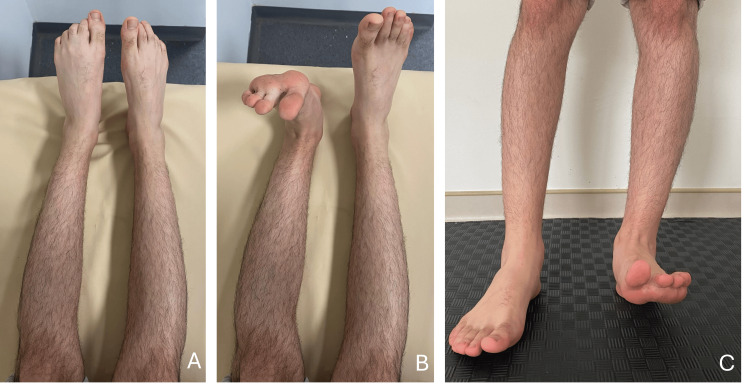
Clinical assessment of a 26-year-old male presenting with acute right-sided foot drop. (A) Plantar flexion remains intact on both sides, with muscle strength graded as 5/5. (B) Ankle dorsiflexion is absent on the affected side (right) in the supine position, with muscle strength graded as 0/5. (C) Standing assessment shows an inability to dorsiflex the right ankle, consistent with foot drop.

A 26-year-old male patient presented with complaints of weakness and numbness in his right foot following prolonged cross-legged sitting on a hard surface for 2-3 hours without changing position. He reported no history of trauma or prior symptoms related to the right lower extremity. He reported that he did not notice any nerve compression symptoms such as tingling, neuropathic pain, heaviness, and numbness of the involved part till he stood up. On physical examination, there was no swelling or ecchymosis in the right knee or ankle, and the range of motion in the joints was full. However, tenderness was noted on palpation at the fibular head. The Tinel test was negative. Muscle strength assessment revealed complete loss of dorsiflexion in the right foot (0/5), while plantar flexion strength was intact (5/5) (Figure 1).

**Figure 2:**
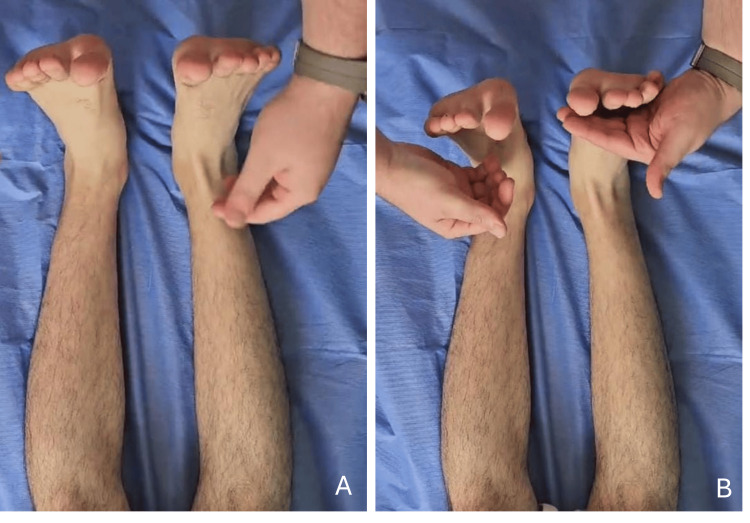
Clinical examination after one month of conservative treatment. (A) Passive dorsiflexion of the right ankle demonstrates normal range of motion. (B) Active dorsiflexion is fully restored, with muscle strength graded as 5/5.

The knee and hip flexor muscle groups were also evaluated as 5/5. The patient was unable to perform heel walking and exhibited hypoesthesia in the first web space of the right foot. The examination of the contralateral lower extremity was unremarkable. To rule out space-occupying lesions, radiographs of the right knee were obtained, showing no pathological findings. The patient was prescribed an ankle-foot orthosis (AFO) to prevent Achilles tendon contracture and falls and was initiated daily oral supplementation, including 500 mg thiamine hydrochloride, 500 mg pyridoxine hydrochloride, 2 mg B12 vitamin, 1,000 mg vitamin C, 1,000 IU vitamin D3, 30 mg zinc, and 300 mg magnesium oxide. Follow-up evaluations were conducted at two weeks and one month. At the two-week follow-up, the AFO was removed, and muscle strength in right foot dorsiflexion was noted to have improved to 3/5, though hypoesthesia in the first web space persisted. Continued use of the medical treatment was advised. By the one-month follow-up, the patient’s muscle strength in dorsiflexion had fully recovered to 5/5, and sensory function in the first web space had normalized (Figure 2). He was able to perform heel walking, and the neuropathy had completely resolved.

## Discussion

This current case illustrated the successful conservative management of peroneal nerve palsy resulting from prolonged cross-legged sitting, with complete recovery achieved through appropriate supportive treatment and rehabilitation. Peroneal nerve palsy is the most common entrapment neuropathy of the lower extremity and ranks third in frequency after ulnar and median neuropathies [1]. Foot drops, often associated with numbness in the lower extremity, should always be considered in the differential diagnosis of such presentations [9]. The etiology of peroneal nerve palsy includes both traumatic and non-traumatic factors [10], with local compression at the fibular head being the most frequent cause [2]. The peroneal nerve’s anatomical course makes it vulnerable to compression and injury, as it directly contacts the periosteum of the fibular neck over a 10-mm length and remains subcutaneous for approximately 4 cm [11,12]. Cases of foot drop following prolonged cross-legged sitting have been reported in the literature, particularly in the 1970s, among agricultural workers who sat in this position for extended periods. This condition was historically referred to as "strawberry picker’s palsy" [4,13]. Foot drop is the most common symptom associated with peroneal nerve palsy, though its exact incidence remains unclear [7]. In cases of atraumatic and sudden-onset foot drop, space-occupying lesions such as synovial cysts or ganglion cysts should be considered [5]. Although rare, compression neuropathies caused by ganglion cysts are most commonly associated with the peroneal nerve [14]. On physical examination, patients with foot drops exhibit an abnormal gait pattern called steppage gait, characterized by the absence of ankle dorsiflexion, which disrupts both the stance and swing phases of gait. Additionally, weakness in ankle eversion may occur due to the involvement of the peroneal muscle groups innervated by the superficial peroneal nerve [6]. In our case, gait analysis performed in our clinic’s gait laboratory revealed a steppage gait, and the strength of the ankle evertor muscles was 0/5. Clinically, involvement of the anterior compartment is more pronounced than the lateral compartment, with the extensor hallucis longus (EHL) being the most affected muscle and the last to recover [15]. In our patient, at the two-week follow-up, dorsiflexion strength was 3/5, while EHL dorsiflexion was absent. By the one-month follow-up, both ankle dorsiflexion and EHL function had fully recovered, consistent with the literature. The Tinel test, which involves percussing the fibular head, is useful in the clinical evaluation of peroneal nerve palsy [6]. In our patient, the Tinel test was negative. Seddon's classification of nerve injury includes three types: neurapraxia (involving myelin injury), axonotmesis (involving myelin and axon injury), and neurotmesis (involving myelin, axon, endoneurium or even perineurium, and epineurium injury) [16]. Electrophysiological studies are helpful in diagnosing and differentiating entrapment neuropathies; however, in the absence of axonal damage, electromyography (EMG) results may be normal in the early stages. Denervation potentials typically appear two to three weeks after injury, and early studies may yield false-negative results [17,18]. Additionally, magnetic resonance neurography (MRN) with T2-weighted and fat-suppression sequences can reveal focal peroneal nerve edema, nerve transections, or muscle atrophy in cases of foot drop [19]. Initial radiographic studies, such as X-rays, are recommended to identify structural changes around the fibular head, including exostoses, tumoral lesions, or osseous pathologies caused by trauma (e.g., chronic arthritic changes or callus formation) [20]. In our patient, two-view radiographs of the right knee ruled out structural abnormalities. EMG was not performed immediately due to the early presentation of symptoms, as acute cases may initially present findings suggestive of false-negative or axonotmesis despite the underlying condition being neurapraxia. Furthermore, in our patient, it was evident that the common peroneal nerve was involved before branching, as symptoms of both the deep and superficial peroneal nerves were observed during the physical examination. The literature includes studies advocating for early surgical decompression to ensure rapid and complete recovery, regardless of the underlying etiology [8]. However, conservative management is the preferred initial approach in most cases, with an emphasis on identifying the underlying cause, planning an appropriate rehabilitation program, and utilizing AFO. Among the conservative treatment options, vitamin B6 has been shown to normalize plasma pyridoxal phosphate levels and alleviate symptoms of peripheral neuropathy within four weeks of use [21]. In line with the literature, conservative treatment was chosen for our patient. This included the use of an AFO, as well as daily oral supplementation with B1 (500 mg), B6 (500 mg), B12 (2 mg), vitamin C (1,000 mg), vitamin D3 (1,000 IU), zinc (30 mg), and magnesium (300 mg as magnesium oxide). At the one-month follow-up, the patient’s neuropathy had completely resolved, with full recovery of muscle strength and sensation in the affected extremity.

## Conclusions

This case highlights a rare presentation of peroneal nerve palsy with foot drop following prolonged cross-legged sitting, where typical nerve compression symptoms were absent until the patient stood up. Peroneal nerve palsy can occur even in the absence of a major trauma or space-occupying lesions, arising from simple causes such as prolonged and fixed sitting positions. Considering that the peroneal nerve is the most commonly entrapped nerve in the lower extremity, it is important to recognize that prolonged, immobile sitting positions (e.g., cross-legged sitting) may lead to nerve palsy. The occurrence of this pathology in the current case without the onset of any nerve compression symptoms such as tingling, neuropathic pain, heaviness, and numbness of the involved part makes it a very rare case scenario. A detailed patient history and thorough physical examination are essential, and close follow-up is required to monitor the progression of neuropathy. The role of medical therapy in conservative treatment remains controversial in the literature. This case demonstrates that peroneal nerve palsy without a major trauma can be completely resolved with careful monitoring and appropriate medical treatment, without the need for surgical intervention. Neurotrophic agents such as B vitamins and vitamin C supplements can support nerve recovery and regeneration. In this case, the use of targeted vitamin and mineral supplementation yielded successful outcomes. However, it is essential to consider the need for decompression surgery in resistant cases. This case underscores the importance of individualized management strategies and highlights the potential for complete recovery with non-surgical interventions in suitable patients. Recognizing such atypical cases can aid clinicians in timely diagnosis and effective management of positional nerve compression syndromes.
